# Effects of uninterrupted dabigatran on the intensity of anticoagulation during atrial fibrillation ablation

**DOI:** 10.1002/joa3.12655

**Published:** 2021-11-19

**Authors:** Takumi Osawa, Hitoshi Mori, Akane Kawai, Daisuke Kawano, Kenta Tsutsui, Yoshifumi Ikeda, Mitsuki Yamaga, Atsushi Sato, Youdou Gatate, Akira Hamabe, Hirotsugu Tabata, Ritsushi Kato, Kazuo Matsumoto

**Affiliations:** ^1^ Department of Cardiology Japan Self Defense Forces Central Hospital Setagaya Japan; ^2^ Department of Cardiology Saitama Medical University International Medical Center Hidaka Japan

**Keywords:** anticoagulation, atrial fibrillation, catheter ablation, dabigatran, uninterrupted anticoagulant

## Abstract

**Background:**

Uninterrupted dabigatran during atrial fibrillation (AF) ablation is now established as the standard therapy. However, there are few reports on the effects of uninterrupted dabigatran on the intensity of anticoagulation during AF ablation.

**Methods:**

We retrospectively analyzed 247 consecutive patients who underwent AF ablation in our hospital from January 2017 to December 2018. Patients who took warfarin or uninterrupted direct oral anticoagulants (DOACs) except for dabigatran were excluded. 89 patients underwent ablation with uninterrupted dabigatran (uninterrupted group, male 71, mean age 59.6 ± 14.0) and 124 with interrupted DOACs (interrupted group, male 105, mean age 56.9 ± 12.9) during AF ablation. The initial ACT level, proportion of ACT levels of more than 300 s, and total amount of heparin were compared. Furthermore, the incidence of procedure complications was also evaluated.

**Results:**

The initial ACT levels were significantly higher in the uninterrupted group, and the total number of ACTs of more than 300 s was significantly higher in the uninterrupted group (uninterrupted vs. interrupted; initial ACT level, 315.6 ± 59.8 vs. 264.5 ± 48.6, *p* < .001; total number of ACTs ≧300, *n* [%], 304/ 484 [62.8 %] vs. 372/745 [49.9%], *p* < .001). The total amount of heparin during procedure was significantly lower in the uninterrupted group (uninterrupted group vs. interrupted group; 12966 ± 4773 vs. 16371 ± 5212, *p* < .001). There was no significant difference in the incidence of complications between the two groups.

**Conclusions:**

In the catheter ablation of AF, uninterrupted dabigatran would be useful to obtain a stable anticoagulation status during the entire procedure.

## Graphical abstract

The ACT levels during the procedure were significantly higher in the uninterrupted group. The percentage of ACT levels of more than 300 during the procedure was significantly higher in the uninterrupted group.

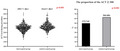

## 1 BACKGROUND

Radiofrequency catheter ablation is an established treatment for symptomatic atrial fibrillation (AF).^1^, ^2^ However, AF ablation has the risk of thromboembolic complications during the perioperative period.^3^ Anticoagulation before and after AF ablation is necessary to prevent these thromboembolic complications. Recently, direct oral anticoagulants (DOACs) have been used to avoid thromboembolic events during the perioperative period of AF ablation. The RE‐CIRCUIT trial demonstrated that uninterrupted dabigatran was associated with fewer bleeding events than uninterrupted warfarin. Based on the American Heart Association and European Society of Cardiology (ESC) guidelines recommend uninterrupted dabigatran during the perioperative period as a Class Ⅰ indication.^1^, ^2^, ^4^ However, the efficacy of uninterrupted dabigatran during the procedure was not well studied. The purpose of this study was to investigate the efficacy of uninterrupted dabigatran for the intensity of the anticoagulant status during the procedure.

## 2 METHODS

### 2.1 Study population

**FIGURE 1:**
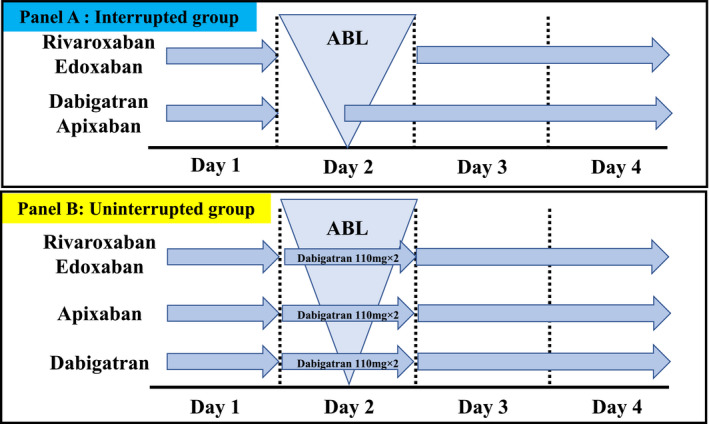
The administration schedule of DOACs during hospitalization. Group A (interrupted group), the anticoagulant medication was interrupted before the ablation. Group B (uninterrupted group), the anticoagulant medication was changed to dabigatran at a dose of 110 mg twice daily on the ablation day and was uninterrupted before the ablation. When patients took a dose of 150 mg twice daily, the administration dose was reduced to 110 mg on the ablation day. ABL, ablation; DOACs, direct oral anticoagulants

We retrospectively analyzed 247 consecutive patients who underwent AF ablation in our hospital from January 2017 to December 2018. The exclusion criteria were as follows: patients who took warfarin and those who underwent the procedure with uninterrupted DOACs except for dabigatran. The patients were divided into two groups; group A (interrupted group), in which the anticoagulant medication was interrupted before the ablation and group B (uninterrupted group), in which the anticoagulant medication was switched to dabigatran at a dose of 110 mg twice daily on the day of the ablation and was uninterrupted before the ablation. When patients took a dose of 150 mg twice daily, the administration dose was reduced to 110 mg on the day of the ablation (Figure 1). This study was performed in accordance with the provisions of the Declaration of Helsinki and local regulations. The patient characteristics are shown in Table 1. The research protocol was approved by the hospital’s institutional review board (02‐003).

**TABLE 1:** Baseline characteristics of the patients

	Uninterrupted group (*n* = 89)	Interrupted group (*n* = 124)	*p*‐value
Clinical parameters			
Age	59.6 (±14.0)	56.9 (±12.9)	.15
Gender, male, *n* (%)	71 (79.8)	105 (84.7)	.35
Height, cm	167.8 (±9.8)	169.9 (±10.2)	.13
BW, kg	69.2 (±13.8)	72.7 (±13.9)	.066
Paf, *n* (%)	64 (71.9)	80 (64.5)	.25
RF ablation, *n* (%)	72 (80.9)	99 (79.8)	.85
Hypertention, *n* (%)	27 (30.3)	32 (25.8)	.47
Diabetes, *n* (%)	9 (10.1)	11 (8.9)	.76
Stroke, TIA, *n* (%)	3 (3.4)	8 (6.5)	.3
CHF, *n* (%)	15 (16.9)	17 (13.7)	.52
CHADS2 score	0.80 (±1.03)	0.71 (±0.99)	.53
Procedure			
Procedure start time, AM, *n* (%)			
AM, *n* (%)	59 (66.2)	87 (70.1)	.55
DOAC			
Dabigatran, *n* (%)	11 (12.4)	10 (8.1)	.28
Edoxaban, *n* (%)	22 (22.5)	30 (24.2)	.93
Rivaroxaban, *n* (%)	50 (56.2)	66 (53.2)	.67
Apixaban, *n* (%)	6 (6.7)	18 (14.5)	.077
Laboratory findings			
UA, mg/dl	6.1 (±1.3)	6.4 (±1.5)	.15
Cr, mg/dl	0.91 (±0.17)	0.91 (±0.19)	.88
aPTT, s	41.2 (±10.3)	40.0 (±8.4)	.35
PT, %	71.7 (±18.6)	74.1 (±16.8)	.33
PT‐INR	1.23 (±0.24)	1.19 (±0.17)	.16
BNP, pg/ml	64.8 (±83.6)	67.2 (±108.7)	.86
TSH, µIU/ml	2.45 (±7.66)	1.68 (±1.63)	.29
fT4, ng/ml	1.11 (±0.13)	1.08 (±0.14)	.17
Echographic findings			
LVEF, %	64.4 (±9.6)	65.2 (±9.2)	.56
LAD, mm	40.8 (±6.5)	41.0 (± 7.2)	.83

Continuous variables are shown as the mean ± SD and categorical variables as the number (%).

Abbreviations: AM, ante meridiem; BNP, brain natriuretic peptide; BW, body weight; CHF, congestive heart failure; DOAC, direct oral anticoagulant; LAD, left atrial diameter; LVEF, left ventricular ejection fraction; Paf, paroxysmal atrial fibrillation; RF, radiofrequency; TIA, transient ischemic attack; TSH, thyroid stimulating hormone; UA, uric acid.

* *p* < .05.

### 2.2 Ablation protocol

All patients took DOACs or warfarin for at least 1 month prior to the procedure. All antiarrhythmic drugs were discontinued for at least 5 half‐lives prior to the ablation. Transesophageal echocardiography was performed to confirm the absence of any left atrial (LA) thrombi before the ablation.

The surface electrocardiogram and intracardiac electrograms were continuously monitored and stored on a computer‐based digital recording system during the AF catheter ablation. All procedures were performed under deep sedation with dexmedetomidine hydrochloride, propofol, and pentazocine. Intravenous heparin was administered at a bolus dose of 100 U/kg immediately after the insertion of all sheaths. Automated ACT measurements were done using the Hemochron Response system (ITC). The ACT was monitored 10 min after the initial heparin infusion and then every 30 min, and additional heparin bolus was administered to maintain an ACT level of more than 300–350 s. The additional heparin was at a dose of 100, 50, or 20 U/kg for ACTs of <250, 250–279, and 280–299 s, respectively.

In the case of RF ablation, ablation was performed with a 3.5 mm tip irrigation catheter (Thermocool SMARTTOUCH^®^; Biosense Webster) under the guidance of a three‐dimensional (3D) mapping system (CARTO3^®^; Biosense Webster). In the case of cryoballoon (CB) ablation, ablation was carried out with the 28 mm/span > second‐generation CB (Arctic Front Advance™; Medtronic) under the guidance of 3D mapping.

### 2.3 Study‐protocol

We compared the ACT level at each timing of the monitored and the number of ACTs less than 300 during the ablation. Furthermore, the incidence of procedure complications such as thromboembolic events, hematomas, and cardiac tamponade was also evaluated.

### 2.4 Statistical analysis

The statistical analyses were performed using JMP software (versionPro14, SAS institute, Cary, CA) and prism (GraphPad Prism9, GraphPad Software inc). Data are expressed as the mean ± SD for parametric data and a comparison of the means between the groups was performed using an independent samples *T*‐test. The categorical data were compared by a chi‐square test. Two‐sided *p*‐values < .05 were considered statistically significant.

## 3 RESULTS

### 3.1 Study population

**FIGURE 2:**
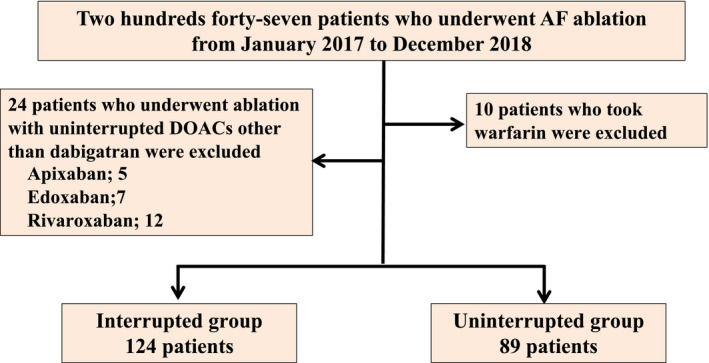
Flow diagram of the patient eligibility. AF, atrial fibrillation; DOACs, direct oral anticoagulants

Figure 2 shows the flow diagram of the patient eligibility. Among the 247 patients, a total of 34 patients were excluded from this analysis. 124 patients were enrolled in the interrupted group and 89 in the uninterrupted group. Baseline patient characteristics are shown in Table 1. There are no significant differences between the two groups in the baseline characteristics, the start time of AF ablations and the type of baseline DOACs uses.

### 3.2 Comparison of the ACT level and proportion of ACT levels of more than 300 s after the initial heparin injection

**FIGURE 3:**
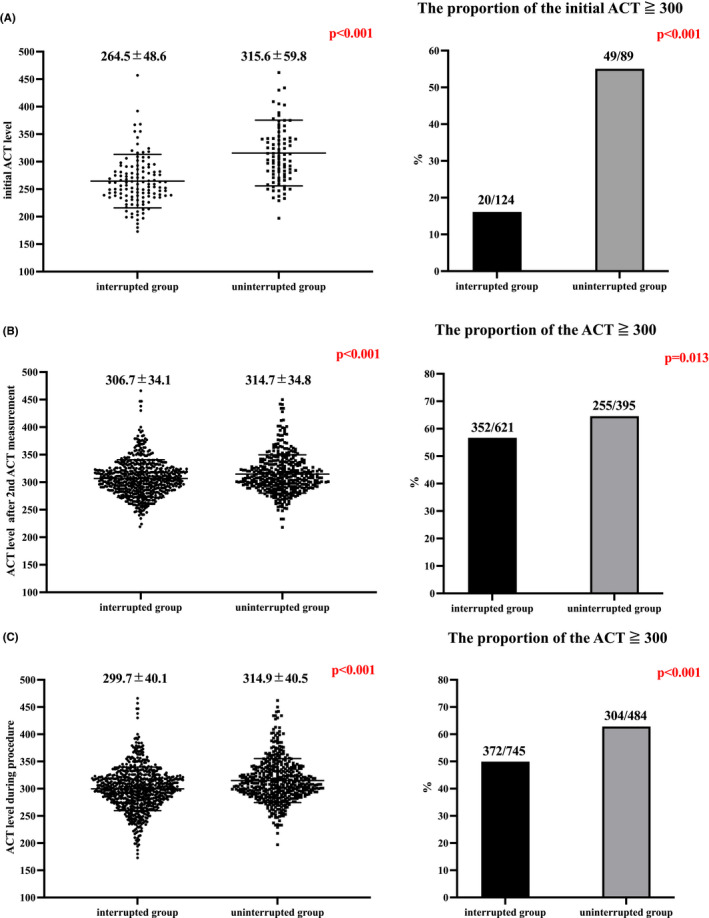
Comparison of the ACT level and proportion of an achievement rate of the target ACT level (≧300 s). (A) Comparison of the ACT level and proportion of an achievement rate of the target ACT level (≧300 s) after the initial heparin injection. The initial ACT levels were significantly higher in the uninterrupted group. The proportion of an initial ACT level of more than 300 was also significantly higher in the uninterrupted group. (B) Comparison of the ACT level and proportion of an achievement rate of the target ACT level (≧300 s) after the second measurements. The ACT levels after the second measurements were significantly higher in the uninterrupted group. The percentage of ACT levels of more than 300 after the second measurements was significantly higher in the uninterrupted group. (C) Comparison of the ACT levels and proportion of the achievement rate of the target ACT level (≧300 s) during the procedure. The ACT levels during procedure were significantly higher in the uninterrupted group. The percentage of ACT levels of more than 300 during the procedure was significantly higher in the uninterrupted group. The continuous variables are shown as the mean value ± SD. ACT, activated clotting time

**FIGURE 4:**
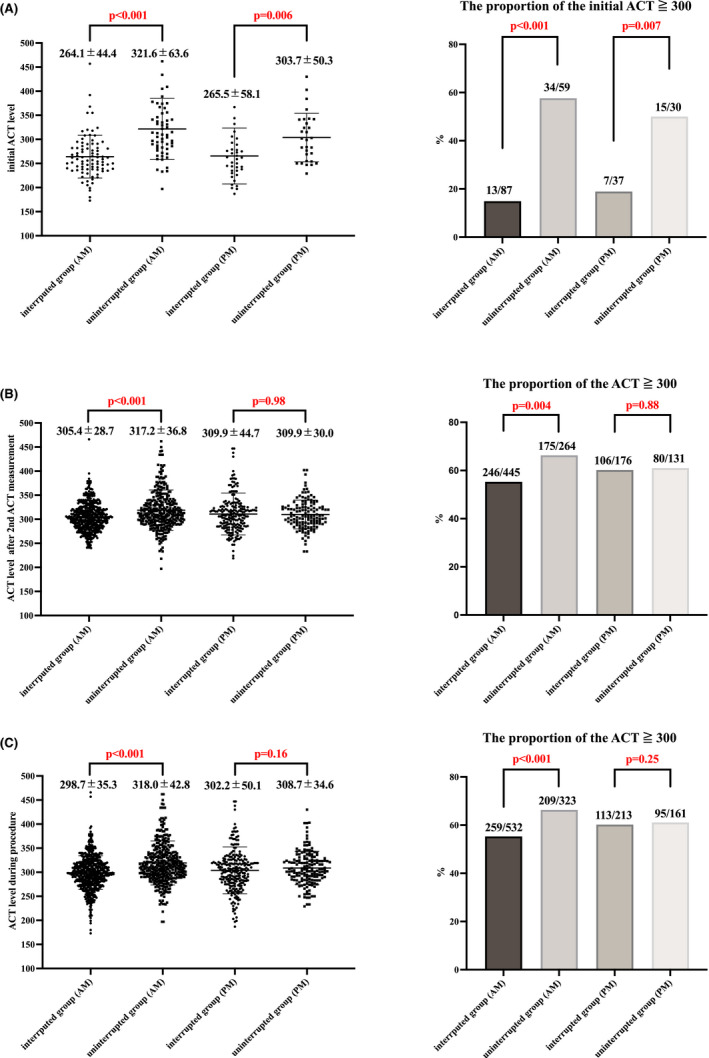
Comparison of the ACT level and proportion of an achievement rate of the target ACT level (≧300 s) for AM and PM. (A) Comparison of the ACT level and proportion of an achievement rate of the target ACT level (≧300 s) after the initial heparin injection for AM and PM cases. The initial ACT levels were significantly higher in the uninterrupted group. The proportion of an initial ACT level of more than 300 was also significantly higher in the uninterrupted group. (B) Comparison of the ACT level and proportion of an achievement rate of the target ACT level (≧300 s) after the second measurements for AM and PM cases. Both were significantly higher in the uninterrupted group for AM cases. However, there are no significant difference between interrupted and uninterrupted groups for PM cases. (C) Comparison of the ACT levels and proportion of the achievement rate of the target ACT level (≧300 s) during the procedure for AM and PM cases. Both were also significantly higher in the uninterrupted group for AM cases. But these have no statistical significance between interrupted and interrupted groups for PM cases. The continuous variables are shown as the mean value ± SD. ACT, activated clotting time; AM, ante meridiem; PM, post meridiem

Figure 3A shows the comparison of the ACT level and proportion of an achievement rate of the target ACT level (≧300 s) after the initial heparin injection. The initial ACT levels were significantly higher in the uninterrupted group (interrupted vs. uninterrupted, sec; 264.5 (±48.6) vs. 315.6 (±59.8), *p* < .001). The proportion of an initial ACT level of more than 300 was also significantly higher in the uninterrupted group (interrupted vs. uninterrupted; 20/124 (16.1 %) vs. 49/89 (55.1 %), *p* < .001). The initial ACT levels were significantly higher in the uninterrupted group regardless of the start time of the procedures (Figure 4A).

### 3.3 Comparison of the ACT levels and proportion of ACT levels of more than 300 s during the procedure

Figure 3B,C shows the comparison of the ACT levels and proportion of an achieving rate of the target ACT level (≧300 s) after the second ACT measurement and the whole procedure. The ACT levels were significantly higher in the uninterrupted group (interrupted vs. uninterrupted, sec; after the second ACT measurement, 306.7(±34.1) vs. 314.7 (±34.8), *p* < .001; whole procedure, 299.7(±40.1) vs. 314.9 (±40.5), *p* < .001). The percentage of ACT levels of more than 300 was significantly higher in the uninterrupted group (interrupted vs. uninterrupted; after the second ACT measurement, 352/621 (56.7%) vs. 255/395 (64.6%), *p* = .013; whole procedure, 372/745 (49.9%) vs. 304/484 (62.8%), *p* < .001). Although the ACT levels were significantly higher in the uninterrupted group, the total amount of heparin during the procedure was significantly lower in the uninterrupted group (interrupted vs. uninterrupted, Unit; 16371 (±5212) vs. 12966 (±4773), *p* < .001). Figure 4B,C shows the comparison of the ACT levels and proportion of an achieving rate of the target ACT level (≧300 s) after the second ACT measurement and whole procedure with cases of ante meridiem (AM) or post meridiem (PM). Although, the ACT levels were significantly higher in the uninterrupted group in AM cases, there were no significant differences in PM cases.

### 3.4 Changes in the mean ACT level during the procedure

**FIGURE 5:**
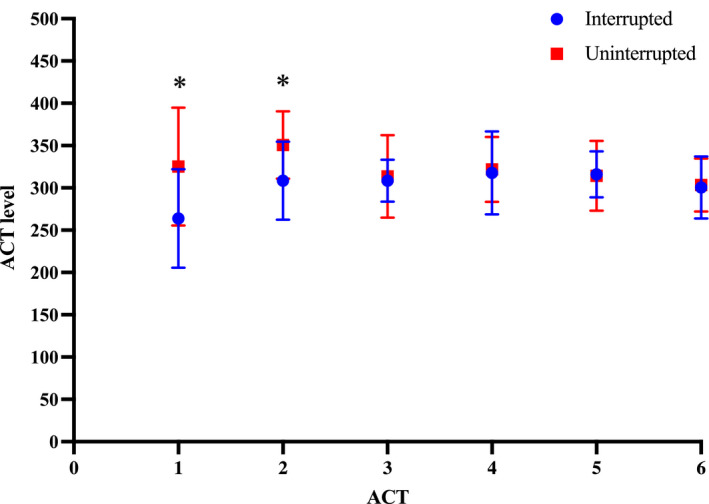
Changes in the mean ACT level during the procedure. The first and second ACT levels were significantly higher in uninterrupted group. The mean ACT level in the uninterrupted group was always higher than that in the interrupted group. The error bar indicates SD. Values are shown as the mean level (* < 0.05). ACT, activated clotting time

Figure 5 shows the chronological changes in the ACT level during the procedure. The first and second ACT levels were significantly higher in the uninterrupted group. Although there were no significant differences other than for the first and second ACT levels, the mean ACT level in the uninterrupted group was always higher than that in the interrupted group.

### 3.5 Perioperative complications

Table 2 shows the perioperative complications. There were no significant differences in the incidence of complications. There were no patients who had ischemic complications. No one needed the use of idarucizumab among the uninterrupted group.

**TABLE 2:** Complications

	Uninterrupted group(*n* = 89)	Interrupted group(*n* = 124)	*p*‐value
Total bleeding complications	1 (1.1)	1 (0.8)	.81
Major bleeding complications			
Cardiac tamponade	0 (0)	0 (0)	NA
Vascular complication	0 (0)	0 (0)	NA
Minor bleeding complications			
Groin hematoma	1 (1.1)	0 (0)	.24
Pericardial effusion without tamponade	0 (0)	1 (0.8)	.40
Thromboembolic complications	0 (0)	0 (0)	NA
Stroke or TIA	0 (0)	0 (0)	NA

Values are *n* (%).

Abbreviations: N/A; not available; TIA, transient ischemic attack.

## 4 DISCUSSION

Our study demonstrated that the continuous administration of dabigatran during the perioperative period of AF ablation provided stable anticoagulant effects during the entire procedure without increasing the bleeding complications. The proportion achieving the target ACT level (≧300 s) was significantly higher in the uninterrupted group, even though the total dose of heparin was significantly low.

### 4.1 The influence of dabigatran on the ACT values

Insufficient anticoagulation during the procedure increases the risk of thromboembolic events.^5^ Hence, it should be recommended to maintain the ACT level at more than 300 s.^6^

More than half of the patients taking dabigatran have prolonged ACT levels beyond the normal range.^7^ Moreover, a higher blood dabigatran concentration increases the ACT level.^8^ The ACT evaluates the function of both the intrinsic and common pathways of the coagulation cascade by measuring the entire blood clotting time. Dabigatran is a direct thrombin inhibitor that blocks the active catalytic site of thrombin (factor Ⅱa) in a competitive and reversible mechanism.^9^ As a result, the higher blood dabigatran level induces a prolonged ACT. On the other hand, the ACT level did not correlate with the concentration of the factor Ⅹa inhibitor and more heparin was needed to achieve the ACT target level in these patients.^10^ In the VENTURE‐AF and ELIMIATE‐AF trials, patients who took rivaroxaban and edoxaban needed significantly more heparin to achieve the target ACT level, as compared to patients who took warfarin.^11^, ^12^ Another study reported that the baseline ACT level in the uninterrupted dabigatran group was significantly longer than that in the rivaroxaban and apixaban group.^13^ Based on our research results, uninterrupted dabigatran may provide more stable anticoagulant activity than factor Xa inhibitors.

Our study also suggested that stable anticoagulant activity was achieved in AM cases, while there was no statistical significance in PM cases (Figure 4A–C). This is because the time between the administration and the start of ablation was longer in PM cases and the effect of DOACs has been weakened.

### 4.2 The utility of a stable anticoagulant during the procedure

Cerebral infarctions after AF ablation have been reported to consist of 0.1%–0.2% symptomatic and 13%–20% asymptomatic.^14^, ^15^ An ACT level less than 250 is a risk factor for perioperative thromboembolism.^7^ Therefore, stable and high intensity anticoagulation would be useful to prevent thrombus formation and reduce the risk of thromboembolic complications. AF catheter ablation with continuous DOACs has a similar frequency of embolic events as that with continuous warfarin.^4^, ^11^, ^12^, ^16^ Also, AF catheter ablation with uninterrupted warfarin reduces the periprocedural embolic events compared to that with bridging with low‐molecular‐weight heparin.^17^ Comparing uninterrupted and interrupted dabigatran therapy during the perioperative period, cerebral embolisms were significantly lower with the uninterrupted dabigatran therapy.^18^ Our study demonstrated that uninterrupted dabigatran can obtain a stable anticoagulant status during the procedure. This stable status might reduce the incidence of ischemic complications.

Dabigatran’s effects can be reversed by idarucizumab, a specific dabigatran‐reversal agent. Even when bleeding events occur, idarucizumab enables a rapid neutralization without any major side effects within minutes.^19^

### 4.3 Study limitations

The present study had several limitations. First, this study was a non‐randomized single‐center retrospective study. So further prospective randomized trials might be needed to validate our results. Second, in our study magnetic resonance imaging (MRI) was not undergone after the ablation. Therefore, the direct influence of uninterrupted dabigatran for the thrombolytic events and the incidence of asymptomatic thrombolytic events was unknown. Third, no one had any major bleeding complications. We could not investigate the safety in the case of major bleeding in the uninterrupted group.

## 5 CONCLUSION

In the catheter ablation of AF, uninterrupted dabigatran would be useful to obtain a stable anticoagulation status during the entire procedure. This might be useful for reducing thromboembolic events during the perioperative period.

## CONFLICT OF INTEREST

The authors declare no conflict of interest for this article.

## AUTHORS' CONTRIBUTIONS

HM contributed to the study conception and design; TO, AK, and HM contributed to the data collection and data analysis; HM and RK contributed to the manuscript revision; and KM provided the study supervision.
